# Entrainment and Synchronization to Auditory Stimuli During Walking in Healthy and Neurological Populations: A Methodological Systematic Review

**DOI:** 10.3389/fnhum.2018.00263

**Published:** 2018-06-26

**Authors:** Lousin Moumdjian, Jeska Buhmann, Iris Willems, Peter Feys, Marc Leman

**Affiliations:** ^1^Institute of Psychoacoustics and Electronic Music, Faculty of Arts and Philosophy, Ghent University, Gent, Belgium; ^2^REVAL - BIOMED Rehabilitation Research Center, Faculty of Medicine and Life Sciences, University of Hasselt, Hasselt, Belgium

**Keywords:** entrainment, synchronization, outcome measures, walking, auditory stimuli, music, cueing, neurological disease

## Abstract

**Background:** Interdisciplinary work is needed for scientific progress, and with this review, our interest is in the scientific progress toward understanding the underlying mechanisms of auditory-motor coupling, and how this can be applied to gait rehabilitation. Specifically we look into the process of entrainment and synchronization; where entrainment is the process that governs the dynamic alignments of the auditory and motor domains based on error-prediction correction, whereas synchronization is the stable maintenance of timing during auditory-motor alignment.

**Methodology:** A systematic literature search in databases PubMed and Web of Science were searched up to 9th of August 2017. The selection criteria for the included studies were adult populations, with a minimum of five participants, investigating walking to an auditory stimulus, with an outcome measure of entrainment, and synchronization. The review was registered in PROSPERO as CRD42017080325.

**Objectives:** The objective of the review is to systematically describe the metrics which measure entrainment and synchronization to auditory stimuli during walking in healthy and neurological populations.

**Results:** Sixteen articles were included. Fifty percent of the included articles had healthy controls as participants (*N* = 167), 19% had neurological diseases such as Huntington's and Stroke (*N* = 76), and 31% included both healthy and neurological [Parkinson's disease (PD) and Stroke] participants (*N* = 101). In the included studies, six parameters were found to capture the interaction between the human movement and the auditory stimuli, these were: cadence, relative phase angle, resultant vector length, interval between the beat and the foot contact, period matching performance, and detrended fluctuation analysis.

**Conclusion:** In this systematic review, several metrics have been identified, which measure the timing aspect of auditory-motor coupling and synchronization of auditory stimuli in healthy and neurological populations during walking. The application of these metrics may enhance the current state of the art and practice across the neurological gait rehabilitation. These metrics also have current shortcomings. Of particular pertinence is our recommendation to consider variability in data from a time-series rather than time-windowed viewpoint. We need it in view of the promising practical applications from which the studied populations may highly benefit in view of personalized medical care.

## Introduction

Research on music and brain typically draws on a cognitive science perspective, in which brain science, psychology, musicology, engineering, and neuroscience (Levitin and Tirovolas, 2009) form the interdisciplinary core for acquiring new insights. While auditory-motor couplings have been studied from a cognitive science perspective, their full potential for clinical applications is not yet fully understood. Yet, evidence-based research related to auditory-motor coupling does hold the prospect of new therapeutic applications in the clinical domain, for example in persons with neurological cognitive or motor impairments. Therefore, in this review, our interest is in the scientific progress toward understanding auditory-motor coupling in rehabilitation and facilitation of walking. Specifically, we look into the process of entrainment and synchronization; where entrainment is defined as the process that governs the dynamic alignments of the auditory and motor domains, whereas synchronization is defined as the stable maintenance of timing during auditory-motor alignment. (In depth explanations of these concepts are followed below).

Meanwhile, the use of music and auditory stimuli for different populations has been studied in people with traumatic brain injury (Bradt et al., 2010), neurological diseases (Moumdjian et al., 2017; Sihvonen et al., 2017), cognitive function in elderly (Li et al., 2015) and dementia (Fusar-Poli et al., 2017; van der Steen et al., 2017). For example, as mobility impairments are prominent in persons with Parkinson's disease (PD) (Marras et al., 2002), evidence has been accumulated that the use of auditory stimuli in rehabilitation for PD patients could improve gait and facilitate walking. At a mechanistic level, several facilitation mechanisms have been suggested, such as the activation of auditory-motor pathways (Thaut, 2015), or the activation effect for the motor system due to the firing rates of auditory neurons which entrain firing rates of motor neurons (Rossignol and Jones, 1976). Clinically, this has led to the development of a technique called Rhythmic Auditory Stimulation (RAS), which generalizes the idea of using auditory stimuli (mainly metronome ticks, but also music) for gait rehabilitation in pathologies of PD (Wittwer et al., 2013), stroke (Yoo and Kim, 2016), and multiple sclerosis (Shahraki et al., 2017). The quality of evidence for using RAS to enhance gait is established by systematic reviews and meta-analysis's on persons with stroke (Nascimento et al., 2015; Yoo and Kim, 2016), cerebral palsy (Ghai et al., 2017), PD (Spaulding et al., 2013; Ghai et al., 2018), and aging population (Ghai et al., 2017). It is likely that these studies provide the foundation for future applications in the respective domains.

However, a closer look at the studies reveals that different gait-related outcomes (e.g., velocity, step length, cadence, etc.) have been used to map out the positive benefits of RAS on gait (Nascimento et al., 2015; Ghai et al., 2017). One may question whether the use of these gait-related outcomes provides enough detailed information about the effects of using RAS or other types of auditory stimuli on gait, specifically within the neurological population with impairments and often asymmetries.

Consequently, at the conceptual level, convergence is needed. A major problem is related to the concept of entrainment, that is, a process that governs the alignment of the auditory and motor domain. This alignment can be understood in terms of coupled oscillators that achieve synchronization by locking into each other's period and/or phase (Bennett et al., 2002; Leman, 2016), or, alternatively, as the effect of minimizing prediction errors (Clayton et al., 2005; Repp and Su, 2013; Leman, 2016). The first is more based on mechanical pull and push forces, while the second is more based on principles of anticipation, involving the concept of an internal model in the brain (Wolpert et al., 1995).

For our purpose, it is straightforward to conceive the interaction between music (or repetitive auditory stimuli) and a person (doing repetitive movements) as a coupled oscillatory system. The beats found in the music (or auditory stimuli) and the footfalls generated by a gait cycle thereby mark the cycles of the two different oscillatory systems. Through entrainment, the beats and the footfalls get aligned in time. That is, the beat and the footfall are constantly pulled and pushed toward one another until the time difference between the beat and the footfall becomes (more or less) stable. From that moment on, the interaction reaches a state of synchronization. This state can be conceived as a dynamic attraction point where the timing differences between music and person are stabilized. Rather than pull and push forces, it is also straightforward to assume error-prediction minimization as mechanism for entrainment (Repp and Su, 2013). For an in depth explanation on the factors that determine the strength of the coupling and entrainment, the reader is referred to Leman (2016). Importantly, the synchronization of the oscillators, which one can view as an outcome of entrainment involving a stable maintenance of timing, can be quantified.

In this review, we focus on the outcome measures that have been used in studies that use entrainment and synchronization as a factor in walking rehabilitation and facilitation. So far, there is evidence that entrainment can be quantified by measuring timing (Repp and Su, 2013). We hypothesize that the use of metrics that measure timing during entrainment and synchronization is beneficial, as it would facilitate our understanding of the mechanisms of the coupling between human gait and auditory stimuli. This understanding can be beneficial as well as enriching for the discipline of rehabilitation. Having access to and understanding of novel assessment measures may contribute toward the development of tailored clinical interventions that use auditory stimuli in neurological gait rehabilitation.

To our knowledge, this work is the first to systematically review the literature in view of the metrics that measure entrainment and synchronization responses to auditory stimuli, during walking in healthy, and pathological populations. The goal of the review is to describe (i) the types of the auditory stimuli, conditions, and the rationale why they were applied, (ii) the metrics which measure entrainment and synchronization to auditory stimuli, (iii) the methods of walking and how they were measured, (iv) the population of participants included in the studies and their motor and/or cognitive characteristics, and finally (v) recommendations for the use of metrics in future research activities.

## Methodology

This review is registered in PROSPERO (registration number: CRD42017080325). We included cross-sectional studies (e.g., observational studies or controlled trials) that consisted of at least one session intervention. The selection criteria for the included studies were adult populations, with a minimum of five participants, investigating walking to an auditory stimulus, with an outcome measure of entrainment and synchronization. Additionally, articles on cyclic activities other than walking were excluded, as well as animal studies, conference proceedings, reviews, and non-English publications.

Two electronic databases (PubMed and Web of Science) were searched up to 9th of August 2017. The following search term were used: Synchronization AND (Rhythm OR Pulse OR Music OR Metronome OR Melody OR Beat OR auditory stimuli) AND (Gait OR Walking OR Treadmill Walking OR Indoor Walking OR Outdoor Walking). Appendix 1 in Supplementary Tables shows the flow of the search strategy. Furthermore, the reference lists of the selected articles were scanned for relevant additional literature.

Two independent reviewers (I.W., J.J.) screened the articles systematically. A third reviewer (L.M.) was contacted in case of disagreements or doubts whether to include a study, and a final decision was made.

**Figure 1:**
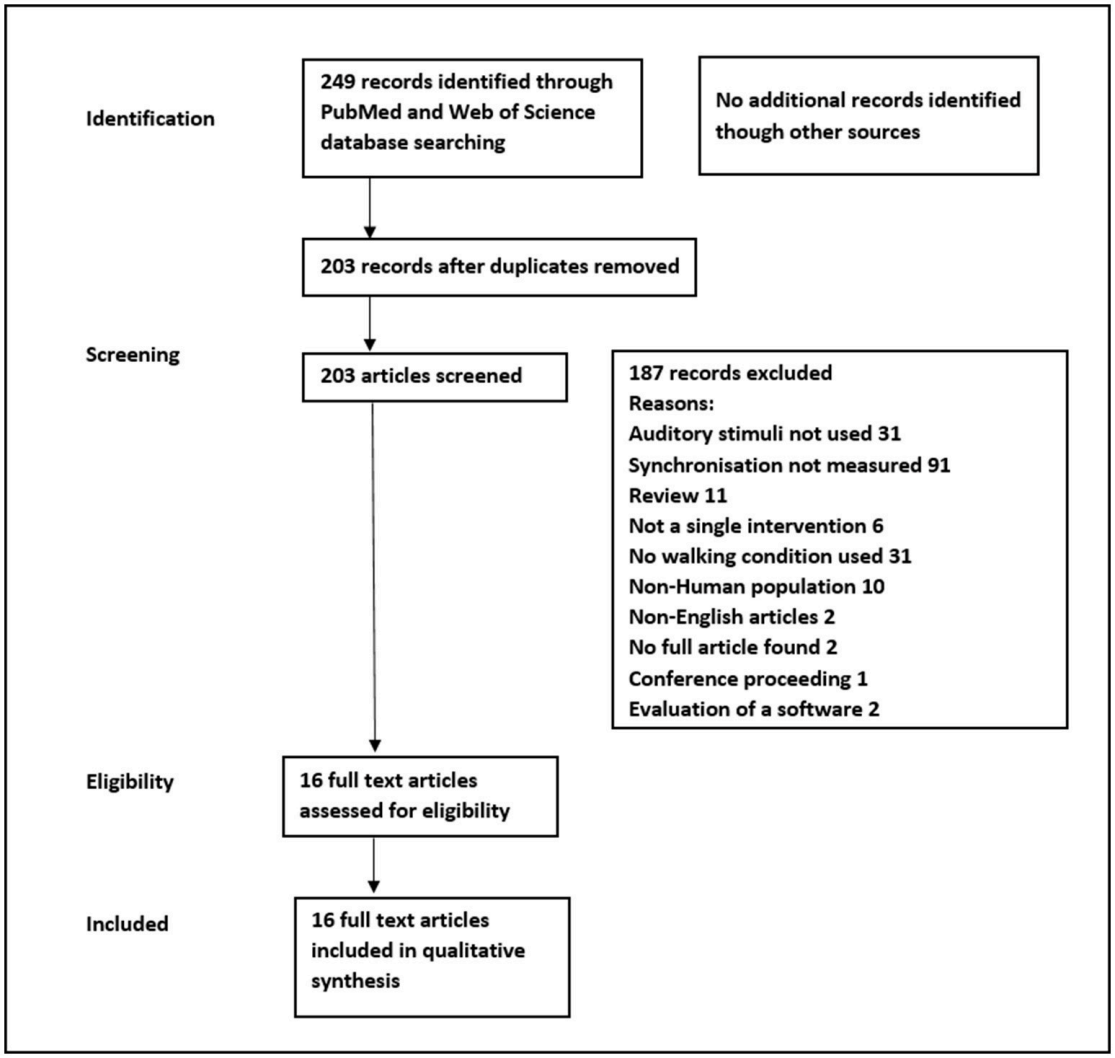
PRISMA flowchart of study selection process.

In total, 16 of the 249 screened articles are included in this review. Figure 1 shows the PRISMA (Liberati et al., 2009) flow diagram summarizing the selection process and reasons for exclusion of the studies. The following data were extracted from the selected studies: participant population (healthy or neurological disease), descriptive characteristics of the participants (age, gender, weight, height), type of pathology in the neurological population (motor and/or cognitive characteristics), number of participants, auditory condition, methods, and equipment used to apply the auditory conditions, experimental and control groups, methods and equipment measuring the walking, outcome measures of entrainment and synchronization, and spatiotemporal parameters.

In order to assess the risk of bias in individual studies, we employed the STROBE checklist (von Elm et al., 2007). To minimize publication biases, the key words from the titles and last author of the included studies were checked for presence on the EU clinical trial register. The planned method of analysis for this review is a descriptive synthesis.

## Results

Below, we present the results of this review in five sections in order to provide a comprehensive methodological overview. The order is presented as such: (i) the risk of bias, (ii) descriptive characteristics of study participants, (iii) the auditory stimuli and/or interactive auditory software, (iv) the parameters and measures of entrainment and synchronization, and finally (v) the sensor equipment and the spatiotemporal parameters of walking across the studies.

### Quality assessment

The quality assessment of the included articles is based on the STROBE checklist. The Supplementary Table shows the results of the STROBE checklist across the included studies. Overall, the quality of the studies was acceptable. All articles had a clear explanation of their scientific background and provided clear explanations of the aims, hypothesis, and experimental design of their study. However, none of the studies provided analysis of the sample size. In four of 16 studies, missing data was not addressed. Strengths and limitations of the studies are addressed in discussion.

### Descriptive characteristics of study participants

Sixteen articles were included in this study. Of the 16 included studies, eight had healthy controls as participants (*N* = 167, age range 17–77; Roerdink et al., 2011; Dickstein and Plax, 2012; Terrier and Deriaz, 2012; Leow et al., 2014, 2015; Marmelat et al., 2014; Mendonça et al., 2014; Buhmann et al., 2016a); three of the 16 included studies had neurological diseases such as Huntington's (Thaut et al., 1999) and Stroke (Pelton et al., 2010; Cha et al., 2014; *N* = 76, age range 31–91) and five of the 16 included studies included both healthy participants and neurological diseases such as PD (McIntosh et al., 1997; Hove et al., 2012; Nomura et al., 2012; Dotov et al., 2017) and stroke (Roerdink et al., 2009; *N* = 101, age range 37–78; *N* = 75, age range 39–79, respectively). See Table 1 for a detailed overview of the descriptive characteristics of participants across studies.

**Table 1:** Descriptive characteristics of participants across studies.

**Article**	**Population**		**Pathology type**	**Sample**	**Male/Female**	**Mean age ± SD**	**Range**	**Participant Characteristics**

	**Healthy subjects**	**Pathology**						
Buhmann et al., 2016a	X		N/A	30	9/21	36.57 ± 14.94	17–77	66.67% musical education; 1/3: music while jogging
Dickstein and Plax, 2012	X		N/A	10	0/10	24.2	22–29	/
Leow et al., 2014	X		N/A	43	19/24	U	18–20	/
Leow et al., 2015	X		N/A	11	5/6	22	U	Music experience: 5.09 ± 6.02 years
Marmelat et al., 2014	X		N/A	Exp 1: 12Exp 2: 12	Exp1: 5/7 Exp2: 7/5	28 ± 6	U	/
Mendonça et al., 2014	X		N/A	9	6/3	25	U	/
Roerdink et al., 2011	X		N/A	20	10/10	63.2 ± 3.6	U	/
Terrier and Deriaz, 2012	X		N/A	20	10/10	36 ± 11	U	/
Cha et al., 2014		X	Stroke	41	24/17	60.8 ± 19.8	U	Onset in months: 6.68 ± 2.35; Able to walk 10 m independently; no hearing; visual deficits; MMSE ≥ 24; 19 R hemiparetic; 22 L hemiparetic
Pelton et al., 2010		X	Stroke	8	5/3	70 ± 12	52–91	Onset >6 months previously; Able to walk 10 m independently; MMSE >21; Average walking speed: 0.81 m/s (0.39); 4 R hemiparetic; 4 L hemiparetic
Thaut et al., 1999		X	Huntington's	27	13/14	47 ± 10.7	31–68	Mean duration of disease: 7 ± 3.3 years; Mean disability score: 1.28 ± 0.65 Mean chorea score: 1.37 ± 0.61
Roerdink et al., 2009	X	X	Stroke	11	7/4	U	42–71	8 R hemiparetic; 3 L hemiparetic; Able to walk 3 min without walking aid
Dotov et al., 2017	X	X	Healthy subjects	10	6/4	U	56–74	/
			Parkinson's	19		U	37–78	H&Y: median stage 2; Median disease duration: 6 years (range: 3–20)
McIntosh et al., 1997	X	X	Healthy subjects	18		U	39–79	/
			Parkinson's (ON)	21	15/6	71 ± 4	U	Mean disease duration: 7.5; H&Y II: 8; H&Y III: 10; H&Y IV: 3
			Parkinson's (Off)	10	6/4	73 ± 3	U	Mean disease duration: 7.8; H&Y II: 4; H&Y III: 6; H&Y IV: 1
Hove et al., 2012	X	X	Healthy subjects	10	4/6	72 ± 5	U	/
			Parkinson's	20	8/12	69.2 ± 7.7	U	Mean duration disease: 3.6 years; H&Y: 2–3; ON medication
Nomura et al., 2012	X	X	Healthy subjects	18	16/2	24.7 ± 2.7	U	/
			Parkinson's	20	8/12	69.2 ± 7.7	U	Mean duration disease: 3.6 years; H&Y: 2–3; ON medication

*N/A, Not applicable; R, Right; L, Left; U, unknown; MMSE, Mini Mental State Examination; H&Y, Hoeln and Yahr; m, meters; m/s , meters/second*.

### The auditory stimuli used across the studies

The type of auditory stimuli used, and the method of administration has been heterogeneous across the studies. Table 2 provides a detailed overview of the applications of the auditory stimuli.

**Table 2:** Descriptive overview concerning applications of the auditory stimuli.

**Article**	**Auditory condition**		**Instructed to synch**		**The experimental condition from an auditory perspective**		**Provided by**	**Study aim**

	**Music**	**Metronome**	**Yes**	**No**	**Pacing frequency (tempo)**	**Condition**		
**HEALTHY PARTICIPANTS** ***(N*** = **167), AGE RANGE 17–77**								
Dickstein and Plax, 2012		X	X		60, 110, and 150 bpm	3 speeds	Metronome signals by a computer	∧
Marmelat et al., 2014		X	X		Co-efficient of variation of 0.5, 1, 1.5, and 2%	Exp1: isochronous metronome at 4 pacing conditions	Metronome signals by a computer	∧∧
					Fractal scaling of H0.2 H0.5, H0.6, and H0.9	Exp2: fractal metronome at 4 fractal scaling conditions		
Roerdink et al., 2011		X	X		77.5, 85, 92.5, 100, 107.5, 115, and 112.5% (of PWS)	7 speeds	Metronome signals by a computer	∧
Terrier and Deriaz, 2012		X	X		x0.7, x1.3 of PWS	2 speeds	Electronic metronome	∧
Buhmann et al., 2016a	X			X	PWS	3 conditions of music: activating, neutral and relaxing music	D-jogger*	∧∧∧
Leow et al., 2015	X	X	X		+15% of PWS	1 speed	Audacity (Free software Inc., Boston, USA) and Beatroot	∧∧∧
Leow et al., 2014^28^	X	X	X		+22.5% of PWS	2 conditions: 20 min, 3 trials each of high groove and low groove music	Audacity (Free software Inc., Boston, USA)	∧∧∧
Mendonça et al., 2014^30^	X	X		X	PWS, +5, +10,−5,−10% of PWS	5 speeds	Matlab generated script	∧
**PARTICIPANTS WITH NEUROLOGICAL DISEASES (*****N*** = **76), AGE RANGE 31–91**								
Cha et al., 2014		X	X		PWS,−10%, +10%, and +20% of PWS	4 speeds	Electronic metronome	∧
Pelton et al., 2010		X	X		/	5 100-pulse trials: each trial consisted of 20 metronome pulses without phase shift at baseline, The phase shifts were positive shifts of 20% of the inter-pulse interval, followed by 4 sections of 20 pulses with one phase shift occurring at an unpredictable time	Audible metronome pulses	∧∧
Thaut et al., 1999	X	X	X		−10%, +20% of PWS	2 speeds	Electronic metronome and MIDI	∧
**CASE-CONTROL STUDIES (*****N*** = **101) AGE RANGE 37–78; HEALTHY CONTROLS (*****N*** = **75), AGE RANGE 39–79**								
Roerdink et al., 2009		X	X		PWS	1 speed with a positive and negative shift phase of 60°	Computer-produced rhythmic acoustic pacing	∧∧
Hove et al., 2012		X		X	U	3 conditions: WalkMate**, fixed RAS tempo and silent control	Matlab generated scripts to control for tempo shifts	∧
Nomura et al., 2012		X		X	U	3 conditions: WalkMate**, fixed RAS tempo and silent control	Matlab generated scripts to control for tempo shifts	∧
McIntosh et al., 1997	X			U	PWS and +10% of PWS	2 speeds of instrumental music in Renaissance style in 2/4 m	synthesizer/sequencer	∧∧
Dotov et al., 2017	X	X		X	+10%	Three variability conditions: no variation, biological variation (long range correlation), non-biological variation (random)	Music: 4 highly familiar musical marches, and amplitude modulated noise	∧∧

*Exp, Experiment; PWS, preferred walking speed; U, Unknown; MIDI, Musical Instrument Digital Interface.Legend of study aims: Change in speeds affecting movement^∧^, Variability in stimuli affecting movement^∧∧^, Influence of musical characteristics on synchronization^∧∧∧^*.

### The custom-made interactive auditory software used across the studies

Two custom made software's were found in the included studies, these were the D-Jogger and the Walk-Mate.

*The D-Jogger* (Moens et al., 2014) is a music player system that adapts the period and phase of a musical playback in response to human interaction. First, the music player identifies the period and phase of a walking or a running person, using the footfall instant as a salient measurement moment. Based on the selected alignment strategy, music is provided, and adapted if needed, using the musical beat as a salient measurement moment. The system consists of a laptop, sensors, headphones, a wifi connection, and transmitter (to transmit the sensor data to the laptop) and an annotated music library (Buhmann et al., 2016b). The reader is referred to the paper of Moens et al. (2014) for a detailed explanation of the components and functioning of the system.

The *Walk-Mate system* (Miyake, 2009) is a human–robot interaction system based on mutual entrainment of walking rhythms. It was developed to investigate the mechanism of interpersonal synchronization, and its potential applications to provide walking support for patients with gait disturbance. The system consists of the following equipment: a laptop, headphones, pressure sensors, a radio transmitter, and receiver (to transmit the sensor data to the laptop). The reader is referred to the paper of Miyake et al. (Miyake, 2009) for a detailed explanation of the components and functioning of the system.

### Measures of entrainment and synchronization

**Figure 2:**
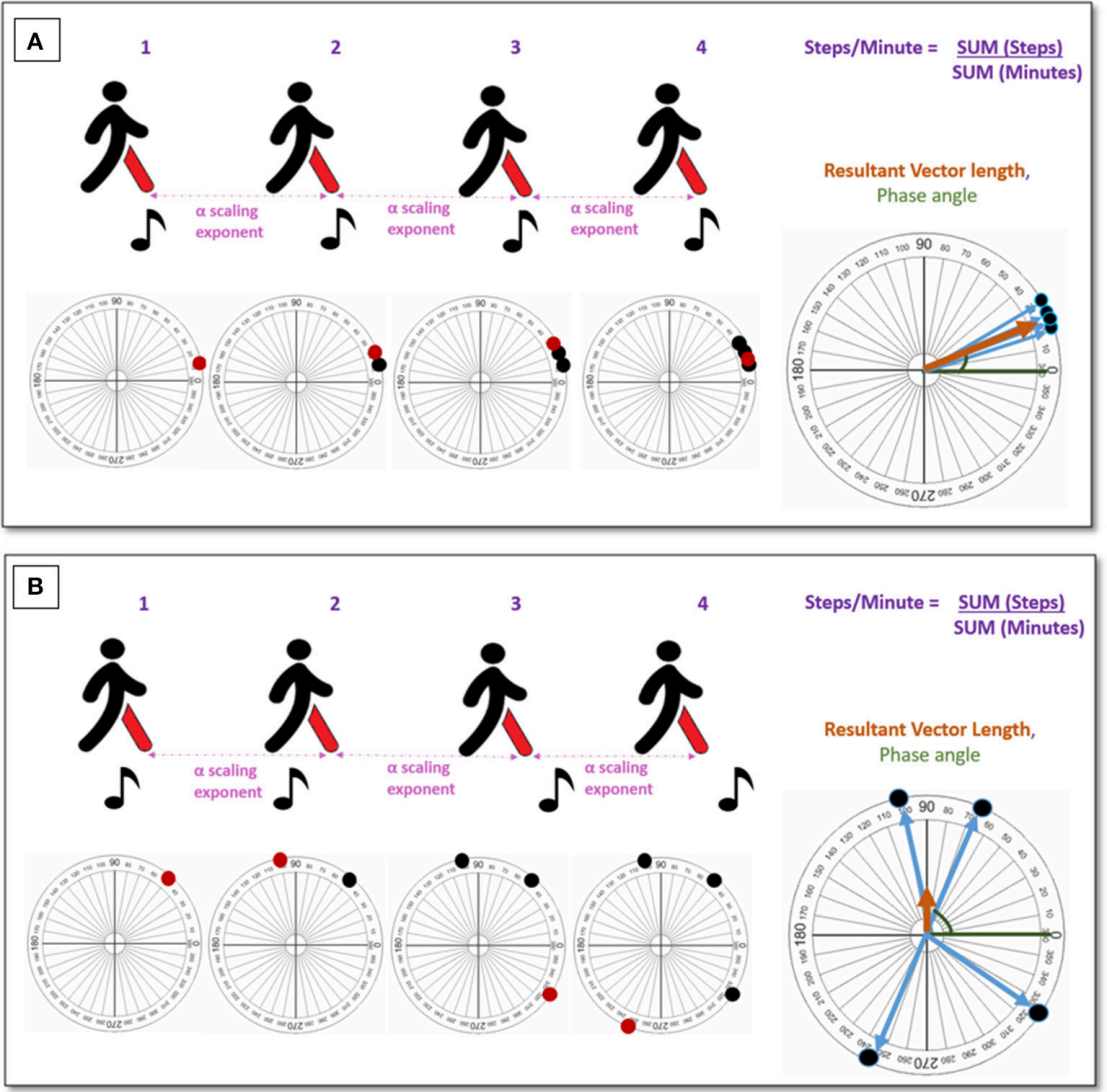
An illustration to measure auditory-motor coupling during entrainment. **(A)** Demonstrates a strong auditory-motor coupling. **(B)** Demonstrates a weak (non-existent) auditory-motor coupling.

Table 3 and Figure 2 provide the definitions, formulas, and interpretations of the below measures of auditory-motor coupling and synchronization during walking. Please note, in our results, we did not find metrics that measure entrainment specifically, but mostly they measure auditory-motor coupling and/or synchronization. In order for correct use of terminology, from this point forward in the text we use the term auditory-motor coupling and synchronization metrics instead of entrainment and synchronization metrics.

**Table 3:** Measurements of auditory-motor coupling and synchronization during walking: definitions, formulas, and interpretations.

**Measurements of auditory-motor coupling and synchronization**	**Definitions, explanations and interpretations**
Tempo *(Beats or steps per minute)*	Tempo is a term that refers to the basic tempo of audio or movement and is typically expressed in number of steps or beats per min (SPM/BPM). SPM is calculated as the total number of steps divided by duration expressed in minutes: $SPM\;=\;\frac{Sum\left(steps\right)}{Sum\left(minutes\right)}\text{Tempo matching occurs if}:\;SPM=BPM$
Relative phase angle*(measured in degrees)*	This is a measure of the timing of the footfall relative to the closest beat. The relative phase angle can be expressed as either a positive (footfall after the beat) or a negative (footfall before the beat) angle in degrees. With the formula below, the relative phase angle for 1 step is calcuated. S_t_ represents the time point where the step investigated takes place, and B_n_ is the beat at the time prior to the S_t_. $\phi =360*\left(\frac{S_{t}-B_{n}}{B_{\left(n+1\right)}-B_{n}}\right)$ To calculate the average relative phase angle, circular statistics (Berens, 2009) is then applied.
Resultant vector length *(expressed as a value from 0 to 1)*	This measure expresses the coherence or stability of the relative phase angles over time. If the distribution of the relative phase angles over time is steep, it results in a high resultant vector length (max value 1). If the distribution of the relative phase angle over time is not steep but broad or multimodal, it results in a low resultant vector length (min value 0). Consider S as a step and n as the *n*th step in the following formula: $\left|R|=|\frac{1}{N}\overset{N}{\underset{n\;=\;1}{\sum }}e^{i\phi S_{n}}\right|$
Asynchrony*(measured in ms)*	This parameter is a measure of the timing expressed in milliseconds (ms) between the footfall and beat instants, i.e., the *asynchrony* between the beat and the footfall. While the phase angles express the *relative* differences between the steps and beats, the intervals between the steps and beats are *absolute differences*. In the below formula, *S_*t*_* represents the time point where the step investigated takes place, and *B_*n*_* is the beat at the time closest to the *S_*t*_*. *asynchrony* = *S*_*t*_−*B*_*n*_
Tempo matching accuracy*(measured in ms)*	This parameter indicates the extent to which the overall tempo of the footfalls matches the overall tempo of the beats. Inter-beat deviation (IBD) was defined as a parameter that measures the tempo-matching accuracy, as expressed by the formula below, where n represents the *n*th step or beat. $IBD=\frac{1}{N}\overset{N}{\underset{n\;=\;2}{\sum }}\left(\frac{\left(B_{n}-B_{\left(n-1\right)}\right)-\left(S_{n}-S_{\left(n-1\right)}\right)}{B_{n}-B_{\left(n-1\right)}}\right)$ The standard deviation of the IBD can also be calculated as a unit of variability of the tempo matching.
Detrended Fluctuation analysis (DFA)*(measured by the scaling exponent alpha)*	The DFA is a common mathematical method to analyse the dynamics of non-stationary time series. More specifically, it characterizes the fluctuation dynamics of the time series through looking into its scaling component alpha (Chen et al., 2002). It has been shown that in other physiological time series the current value possesses the memory of preceding values. This phenomenon is known as long-range correlations, long-term memory, long-range correlations and fractal process of 1/f noise. A healthy gait time series pattern consists of a fractal statistical persistent structure equivalent to a pure 1/f noise (Goldberger et al., 2002). Authors suggest that the analysis of this gives an insight into the neuro-physiological organization of neuro-muscular control and the entire locomotion system (Hausdorff, 2007). The 1/f noise is correlated with a scaling exponent alpha value between 0.5 and 1.0 (indicative of a walking pattern found in healthy gait time series). If alpha is ≤0.5, it signifies an anti-correlation, and is associated with unhealthy walking pattern (randomness). For details of calculating the scaling exponent alpha, the reader is referred to Chen et al. (2002) and Terrier et al. (2005). The underlying rationale of using this analysis method in gait is addressed in the discussion section of our review. The integrated time series of *N* is divided into boxes of equal length. Each box has a length “*n*” and in each box of length *n*, a least square line is fit on the data.
	The y- coordinate of the straight line segments is denoted by *y_*n*_(k)*. The integrated time series *y(k*) is detrended by subtracting the local trend *y_*n*_(k)* in each box. The root mean square fluctuation in this integrated and detrended time analysis is calculated by: $F\left(n\right)=\sqrt{\frac{1}{N}\overset{N}{\underset{k\;=\;1}{\sum }}{\left[y\left(k\right)-y_{n}\left(k\right)\right]}^{2}}$ Thus, the fluctuations can be categorized by the scaling exponent (ALPHA), which is the slope of the line relating Log*F*(*n*) to log(*n*):
	*F*(*n*)∽*n*^α^

In the included studies, six parameters were found to capture the interaction between the human movement and the auditory stimuli:

#### Cadence (measured in steps per minute), tempo (measured in beats per minute)

Seven of the 16 included articles used this parameter as a measure of tempo matching (McIntosh et al., 1997; Thaut et al., 1999; Roerdink et al., 2009, 2011; Cha et al., 2014; Mendonça et al., 2014; Dotov et al., 2017).

#### Relative phase angle (measured in degrees)

Five of 16 studies measure the relative phase angle (McIntosh et al., 1997; Roerdink et al., 2009, 2011; Nomura et al., 2012; Buhmann et al., 2016a), while two of these studies report the variance expressed by a standard deviation value as well (Roerdink et al., 2009; Nomura et al., 2012). Additionally, one study used the term *phase coupling* (McIntosh et al., 1997) for referring to this parameter.

#### Resultant vector length (expressed as a value from 0 to1)

Four of 16 articles used this parameter in their study (Hove et al., 2012; Nomura et al., 2012; Buhmann et al., 2016a; Dotov et al., 2017). Of the four studies, one used this parameter in order to group their study population into two categories of phase coherence and incoherence (Buhmann et al., 2016a). A second study used this parameter, yet they used the term *synchronization consistency* (Dotov et al., 2017).

#### Asynchrony (measured in MS)

Three of 16 studies used this parameter (Pelton et al., 2010; Roerdink et al., 2011; Dickstein and Plax, 2012). Of the three studies, one calculates the variability of the timing as well (Roerdink et al., 2011).

#### Period (or tempo) matching performance (measured in MS)

Two studies from the same research group (Leow et al., 2014, 2015) calculated the period matching performance through the inter-beat interval deviation. They defined the inter-beat deviation as a parameter that measures the tempo-matching accuracy. They also calculate the standard deviation of the inter-beat deviation, and name this parameter the tempo-matching variability (Leow et al., 2015). The third paper measuring the period matching performances does so by calculating the proportion asynchrony error which is relative to the target pulse (also described as period control; Pelton et al., 2010). A fourth paper does this by calculating an error measure *E* to evaluate the frequency error of the synchronization. When *E* = 0, there was a perfect frequency synchronization. When *E* was negative, the participants needed to take more strides before synchronizing (Roerdink et al., 2009). This parameter was also referred to as *period control*.

#### Detrended fluctuation analysis (DFA) (measured by the scaling exponent alpha)

Three (Hove et al., 2012; Terrier and Deriaz, 2012; Marmelat et al., 2014) of the 16 studies included in this review use this analysis for slightly different purposes. One study investigated if the use of an interactive music player (one that adjusts its timing of the beats to the timing of the footfalls) retains the most healthy gait speed. That is one that equals the long range correlation of a healthy speed in Parkinson patients (Hove et al., 2012).

The remaining two studies used this parameter to measure how long-term correlations of gait in a healthy population were influenced when changes of the experimental conditions were imposed on healthy controls. Their main research questions were, how long-term correlations change when using isochronous, non-isochronous, and fractal cues (Marmelat et al., 2014), and how these correlations change when imposing simultaneous variations of speed and rhythm (using a treadmill and metronomes; Terrier and Deriaz, 2012).

### The sensor equipment used across the studies

Various sensors were used to capture the movement parameters. The spatiotemporal parameters of gait that were calculated using the described sensor equipment are summarized in Table 4.

**Table 4:** A summary of spatiotemporal gait parameters, auditory-motor coupling, and synchronization parameters measured across studies.

	**Auditory-motor coupling and synchronization measures**						**Gait parameters**								
**Articles**	**Resultant vector length**	**Relative phase angle**	**Relative timing**	**Period matching**	**DFA**	**Cadence**	**Stride length**	**Velocity**	**Step length**	**Double support time**	**Stride width**	**Stride speed**	**Stride time**	**Double limb support**	**Gait asymmetry**
	**(a.u.)**	**(**°**)**	**(ms)**	**(ms)**	**(**α**)**	**(spm)**	**(m)**	**(m/s)**	**(m)**	**(%)**	**(m)**	**(m/s)**	**(s)**	**(%)**	**(%)**
Dickstein and Plax, 2012			x												
Marmelat et al., 2014					x										
Roerdink et al., 2011		x	x			x									
Terrier and Deriaz, 2012					x		x					x	x	x	x
Buhmann et al., 2016a	x	x					x	x							
Leow et al., 2015				x			x	x					x		
Leow et al., 2014				x			x	x	x	x	x		x		
Mendonça et al., 2014						x									
Cha et al., 2014						x	x	x						x	x
Pelton et al., 2010		x	x	x											x
Thaut et al., 1999						x	x	x							x
Roerdink et al., 2009		x				x			x				x		x
Hove et al., 2012	x				x										
Nomura et al., 2012	x												x		
McIntosh et al., 1997		x				x	x	x							
Dotov et al., 2017	x					x	x								

#### We grouped the sensor technology into four categories:

(a) Portable sensors

Four systems were used across six studies. These included the pressure-sensitive foot switches (Noraxon Ltd.) (Dickstein and Plax, 2012), pressure sensors in the foot (Hove et al., 2012; Nomura et al., 2012), sensors embedded in shoe inserts (infotronic CDG, Eindhoven, The Netherlands) (Thaut et al., 1999), and OPAL IMU sensors (MobilityLab, APDM, Inc., Portland) (Buhmann et al., 2016a; Dotov et al., 2017).

(b) Fixed sensors on a treadmill.

Two different types of sensors were used across three studies: a single large force platform mounted on the treadmill (ForceLink, Culemborg, The Netherlands) (Roerdink et al., 2011; Marmelat et al., 2014) and the FDM-TDL (Scheinworks/Zebris, Schein, Germany). The latter is a treadmill which has 7,168 pressure sensors embedded (1.4 sensors per cm^2^) in its surface (Terrier and Deriaz, 2012).

(c) Sensored walkways

Three types of walkways were used across four studies: a Zeno pressure sensor walkway of 16 foot with a sampling rate of 120 Hz (Leow et al., 2014, 2015); a GAITRite system (GAITRite, CIR systemInc, USA, 2008), with an active area of 366 cm long, containing 16,128 pressure sensors (Cha et al., 2014); and a computerized foot switch system consisting of four separate sensors which measure the surface contact of the heal, toe and 1st and 5th metatarsal (McIntosh et al., 1997).

(d) 3D motion capture

Three different systems were used across three studies: A six camera motion capture (Vicon MX3+, MXF20 at 120 Hz; Mendonça et al., 2014); an Oxford Metrics Vicon tracking system with six infrared cameras which captured the motion of ankle markers at 120 Hz (Pelton et al., 2010) and a 3D passive-marker motion registration system (SIMI motion; Roerdink et al., 2009). In the latter, markers where attached to the heels of the shoe of participants.

## Discussion

In this systematic literature review, we included 16 articles that measured timing components of auditory motor coupling and synchronization while walking to auditory stimuli (metronomes and music). Half of the studies were in healthy subjects exclusively, while the other half also included persons with neurological conditions. Six outcome measures were found: steps per minute, resultant vector length, relative phase angle, relative timing, period matching performance and DFA.

All the metrics we identified, with the exception of the DFA, provide general information about the synchronization of the motor system and the auditory stimuli. Typically, the metrics point to average timing relationships between footfalls and beats, which are the salient markers of the cycles that characterize the essential features of the motor system and the auditory stimuli. In other words, the metrics (with the exception of DFA) can be used to quantify synchronization in an environment where auditory stimuli and gait are coupled. The metrics assume that the best synchronization state is the state where the audio-motor error is minimal, preferably zero. However, it is important to note that the underlying entrainment, which we defined as an alignment dynamics, understood in terms of pull and push forces (mechanics) or audio-motor prediction-error correction (internal models) is not captured by these metrics.

### Did synchronization influence walking?

Seven of the 16 included studies analyzed the effects of different auditory stimuli on gait parameters. That being said, heterogeneity is present in the included studies in terms of the investigations on the different aspects of synchronization to accommodate the different hypotheses and a variety of study aims. Examples of heterogeneity in the methodological applications are the different methods to produce the auditory stimuli, the different stimuli, and characteristics within the stimuli, the use of tempo or phase shifts at different tempi in the experimental conditions, and lastly, the different participant populations in the studies. Similarly, heterogeneity is also found in the reported spatio-temporal parameters of gait.

Given the fact that these heterogeneities are seen in the study designs, this hampers the direct comparison of the results reported in the studies. Accordingly, we are not able to estimate the overall effect of synchronization in gait. Instead, below, we provide a short overview of the effects of synchronization on gait seen per study without any direct comparisons between studies.

#### In healthy participants

Buhmann et al. (2016a) showed that in healthy participants, synchronization was not specifically necessary to obtain changes in spatiotemporal parameters of gait. However, the authors speculated that the auditory-motor coupling in the process of entrainment was still the main source that brought about the changes. In the study of Roerdink et al. (2011), the cadence of healthy participants increased with the imposed pacing frequencies. When the pacing was close to the preferred cadence, the variability or relative timing was diminished. This in turn resulted in higher phase coherence, i.e., it resulted in a higher stability of synchronization.

#### In neurological participants

In the study of Cha et al. (2014) (with stroke patients), higher RAS tempi lead to a faster gait velocity, cadence, and stride length and a reduced double limb support duration on both the affected and unaffected lower extremity. The authors reasoned that with a reduced double limb support, the walking pattern became more stable and therefore the balance was increased. Moreover, they concluded that higher RAS tempi allowed for the stroke patients to have larger stride length on their affected side, compared to the non-RAS conditions.

In the study of Roerdink et al. (2009), both stroke patients and healthy participants were included, and results indicated that pacing with a double metronome (each metronome pulses the pacing of each other step) was better than pacing with a single metronome (one pulse pacing each step) for both patients and healthy controls in terms of decreasing step asymmetry. Lastly, the study of Pelton et al. (2010) with stroke patients (comparing paretic and non-paretic lower extremities) concluded that nor the accuracy nor the variability was altered when walking on a treadmill in the presence of the metronome compared to walking without a metronome. However, the authors noted that these results could be explained by the high level of symmetry that already was observed during treadmill walking. Thus, little room was available for improvement, if any would be seen.

Other neurological pathologies were also found in this review. Thaut et al. (1999) compared walking to music and metronomes for patients with Huntington's disease with three levels of disability. Self-paced, slow, and fast metronomes and music were used. The study demonstrated, that of 27 patients, 19, 23, and 17 were able to increase their velocity from their baseline during the self-paced (no audio), accelerated metronome, and music conditions, respectively. The participants that did have a change in velocity were more disabled. Moreover, the authors also assumed that the difference in numbers for metronome and music might have been caused by the complexity of the music compared to the simple ticking of the metronomes. A crucial finding which the authors comment on, is that the impaired performance in a sensorimotor synchronization task might be a predictor of the neurological disease prior to the evidence of the first symptom in persons with Huntington's disease. Yet, they do emphasize that the general mechanisms for rhythmic entrainment are intact at earlier stages of the disease.

Finally, in PD patients, McIntosh et al. (1997) concluded that a faster RAS condition lead to an increase in velocity, cadence, and stride length. The results were also similar to healthy controls. However, they also report that a high severity of the disease lead to a worse synchronization.

In summary, an assumption can be made that, overall, synchronization to auditory stimuli had a positive effect on the gait of different patient populations. However, many considerations can be taken in order to have a robust answer to that assumption. Below, these are discussed.

#### Limitations of the included studies

One of the major limitations of the studies reviewed here was the small sample size in some of the studies (minimal 9, maximal 12; Pelton et al., 2010; Dickstein and Plax, 2012; Marmelat et al., 2014; Mendonça et al., 2014; Leow et al., 2015). Furthermore, in the studies conducting DFA analysis (Hove et al., 2012; Marmelat et al., 2014), the trials are 3 min, which is a relative short time for the DFA analysis, and longer trials are warranted for future studies (Pierrynowski et al., 2005).

Other limitations of studies were due to missing data as a result of technological errors (Leow et al., 2014). In addition, some studies included patients who did not exhibit motor dysfunctions, for example the PD patients included in Dotov et al. (2017) did not exhibit clinical dysfunctions such as freezing gait (Dotov et al., 2017). In one study, the cognitive impairment of patients was not taken into account, and therefore patients who did not synchronize were excluded without discussing the underlying causes (Roerdink et al., 2009). Moreover, the methodological design can be questioned, as patients could rest as long as they wished, while listening to the next auditory condition, making the exposure time to sounds variable across participants (Roerdink et al., 2009).

### Critical appraisal of the identified metrics

The DFA measure differs from the remaining metrics identified in this review, because the DFA is not a pure measurement of entrainment and/or synchronization *per se*. Rather than saying something about the audio-motor relationship, the DFA provides information about the variability of movement during the process of entrainment and/or synchronization. As known, this variability can be related to the quality of movement (Dotov et al., 2017).

A healthy gait time series pattern consists of a fractal statistical persistent structure equivalent to a pure 1/f noise (Goldberger et al., 2002). Authors suggest that the analysis of this gives an insight into the neuro-physiological organization of neuro-muscular control and the entire locomotion system (Hausdorff, 2007). The 1/f noise structure demonstrates a non-random predictability of the steps in a gait cycle. Alternatively, incorrect time-series that have been found in gait have been associated with various diseases (Goldberger et al., 2002; Hausdorff, 2007). It has been claimed that the loss of the non-randomness (the statistical persistence) could be related to the decreased adaptability of neural structures and looser cortical control (Goldberger et al., 2002; Hausdorff, 2007).

The DFA could potentially be a valuable measure to help explain entrainment and/or synchronization in terms of variability rather than in terms of prediction-error minimization. Such an approach would cope with the theory of active inferencing (Brown et al., 2013; Friston et al., 2017), in which a subject's motor variability is understood as a way to sample audio-motor errors so that statistical inferences about those errors can be more accurate. In other words, according to the active inferencing concept, subjects (due to neuromuscular variability) generate small variations in footstep timing but this variability helps them to better estimate the audio-motor error, so that smooth entrainment and/or constancy in synchronization, despite some variability, can be maintained. According to this theory, the differences seen during movement between healthy subjects and subjects with neurological diseases may point to the ability of handling variability, rather than minimizing prediction error. The theory at least assumes that variability measures may be a crucial factor to be taken into consideration, in addition to the synchronization metrics.

**Figure 3:**
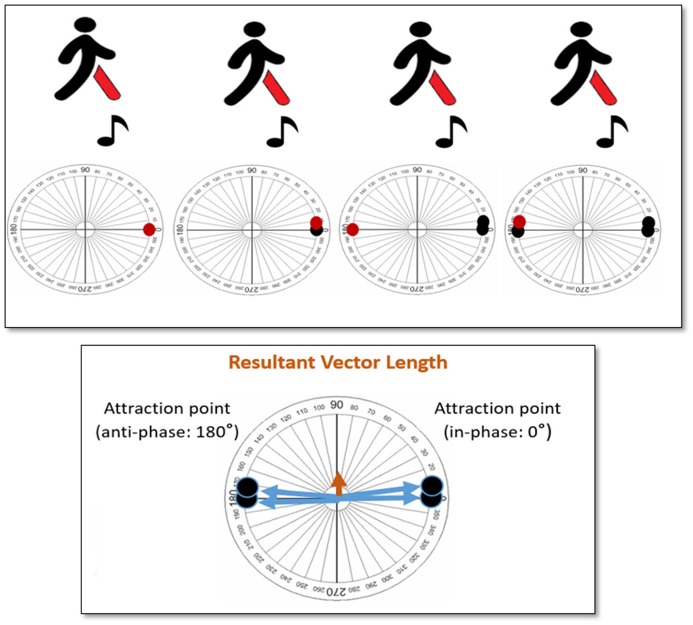
Illustration of a scenario; where the walker entrains his/her steps to the beats in the auditory stimuli, and switches between in-phase (0°) and anti-phase (180°) attractors.

It is also very important to note, that the DFA considers variability from the viewpoint of continuity in time, that is, as a time series. The other metrics identified in this review are based on average values across different time points. Hence, their variability measure is based on time windows, rather than time series. This difference between time series and time window becomes crucial when considering the attraction dynamics that underlie synchronization. As we work with cyclic or oscillatory phenomena in both the motor and audio domain, the state of synchronization can typically be reached by two attraction points, one at in-phase (i.e., footfall and beat occur together) and the other at anti-phase (i.e., footfall and beat occur at 180° difference; Haken et al., 1985; Leman, 2016). To better understand this phenomenon, the reader is directed to Figure 3. In this illustration, we present a scenario of a walker entraining to the beats in the auditory stimuli. This walker reaches an in-phase (attractor point at 0°) synchronization, but changes to an anti-phase (attractor point at 180°) synchronization during the course of the trial. When we calculate the resultant vector length according to the described metric in this review, we end up with a very small value, which seems to indicate that the walker, overall, did not have a cohesive phase synchronization. However, in reality, this is not true; the walker maintains synchronization, overall, but the walker suddenly changed and followed a different attractor across time. To capture this phenomenon, we need methods that describe synchronization as time-varying.

### Practical considerations for the application of auditory stimuli

#### Choosing the auditory stimuli; is it crucial?

Another crucial aspect in designing studies that use music as auditory stimuli may be the choice of music. In our review, we found two studies that discuss the importance of music selection. The study of Buhmann et al. (2016a) distinguished between the activating and relaxing characteristics of the music. They concluded that the stride length was significantly larger when walking to activating music compared to relaxing music. Activating music has been defined as music that has an increasing effect on walking velocity and/or stride length, whereas relaxing music typically decreases these parameters. The acoustical analysis revealed that the activating music has a more binary emphasis patterns (actually matching the alternating footstep pattern), whereas the relaxing music has a more ternary emphasis pattern, where emphasis is present or absent in each three or six beat periods. Similarly, high groove music has also been found to have a positive effect on the stride length, stride time, and velocity compared to low groove music (Leow et al., 2014). Groove is a musical characteristic associated with the clarity of the beat in the music (i.e., beat salience). Groove is defined as the desire to move: the higher the groove, the higher desire to move, and vice versa (Madison, 2006).

#### Synchronization; are instructions needed?

Familiarization sessions have been shown to be important in order to get reliable task performances during experiments. In that context, the task may involve explicit instruction to synchronize to the music, or, alternatively, the task may involve spontaneous (non-instructed) synchronization. In our review, two studies focused on spontaneous synchronization: Mendonça et al. (2014) showed that spontaneous synchronization did not occur without instructions at tempi higher than the participants' natural cadence. Buhmann et al. (2016a) also focus on spontaneous synchronization by providing music with a tempo as close as possible to the walking cadence, in order to induce a spontaneous optimal level of synchronization. To achieve that goal, they used the D-Jogger technology to automatically match the tempo of the music to the walking cadence. Participants were not instructed to synchronize and results showed that approximately half of them walked in optimal synchrony with the musical stimulus whereas the other half lost synchrony to some degree. Instructing to synchronize might have resulted in more synchronized trials. However, the disadvantage of imposing synchronization as task is that it augments cognitive demand, as synchronization can be seen as a supplementary task to the walking task. Such a dual task might be problematic for certain pathologies with cognitive impairments.

### The derivate: an interdisciplinary viewpoint

Given the interdisciplinary nature of the study topic, we believe that a systematic review can provide a helicopter view on methods and data that is beneficial for continued empirical research. Such a viewpoint is beneficial for pinpointing general weaknesses in the overall scientific approach (cf. our discussion about variability).

In addition, such a viewpoint may suggest new interdisciplinary research lines, such as in the domain of neurological rehabilitation. For example, the measures of auditory-motor coupling and synchronization can be used to guide and prompt clinicians and researchers to include the assessment methods and measures of auditory-motor coupling and synchronization in studies investigating deviant gait patterns in neurological populations and the impact of auditory stimuli on it. On its turn, experimental data in patient groups with disordered neural control will contribute to deeper understandings into the different dynamics of the auditory-motor coupling. Foreseeing a complementary view-point, that these auditory-motor coupling and synchronization metrics can perhaps function as a diagnostic tool to assess certain co-ordination qualities in movements of neurological patients with cerebellar dysfunctions such as ataxia. All the above may result in the development of innovative and promising clinical interventions using tailored auditory conditions for neurological gait rehabilitation.

Yet, the inconsistent terminology found in the identified metrics of auditory-motor coupling and synchronization over different studies is problematic. Parameters have many synonyms, but these synonyms may hamper the fluent understanding of some studies in this domain. For example, relative timing, asynchrony, and phase control all refer to the interval between the beat and the foot contact, which is measured in milliseconds. Yet different terms are used and different formulas are used to calculate measured outcomes. An explanation for the non-unanimous terminology could be the lack of standardized equipment. In our review, we traced two hardware-software systems, the D-Jogger (Moens et al., 2014) and the Walk-Mate (Miyake, 2009), but these systems are custom made. The remaining studies used commercially available lab constructed technology, with proper commercial standards. We believe that the differences in terminology for the metrics that address similar outcome measures, may be a consequence of following commercial standards. It is likely that this terminological confusion may hamper interdisciplinary progress, in particular of translating empirical findings across disciplines. The confusion can be narrowed down by coming to a consensus on terminology, as well as a willingness to understand each other's disciplinary terminology by adopting an interdisciplinary viewpoint at the cost of a small (time) effort to learn terminology of other disciplines.

Overall, we believe that the interdisciplinary viewpoint provides a powerful potential to level-up the scientific achievements within the individual disciplines. For example, for the discipline of neurological rehabilitation, understanding the complex parameters of neurodegenerative diseases, as well as having access to calculate and measure these parameters in an efficient way, using a cross-disciplinary consensus on terminology and metrics, will be an enrichment. Coupled with appropriate interpretations, the parameters will provide novel paths to understand clinical dysfunctions from different perspectives and, in turn, advance current clinical practice. In a similar scenario, the interdisciplinary viewpoint offers disciplines, such as musicology, opportunities to study the underlying mechanisms of movement-music entrainment from a neuro-socio-scientific viewpoint of brain, agency, motivation, and expression. This interdisciplinary viewpoint is applicable for many other disciplines (few examples: cognitive sciences, engineering) as well.

## Conclusion

In this systematic review, several metrics have been identified, which measure the timing aspect of auditory-motor coupling and synchronization of auditory stimuli in healthy and neurological populations during walking. The application of these metrics may enhance the current state of the art and practice across the neurological gait rehabilitation. These metrics also have current shortcomings. Of particular pertinence is our recommendation to consider variability in data from a time-series rather than time-windowed viewpoint. A robust community of researchers across different disciplines may be a way to achieve genuine interdisciplinary. We need it in view of the promising practical applications from which the studied populations may highly benefit in view of personalized medical care, assistance, and therapy.

## Author contributions

LM, IW, and PF were involved in the search strategy establishment and application of the methodology of the systematic review. LM and IW extracted the data from the included articles. LM, IW, JB, PF, and ML were involved in formulating the tables, formula's, and figures created in this paper. LM, JB, PF and ML had direct intellectual contribution to the written text.

### Conflict of interest statement

The authors declare that the research was conducted in the absence of any commercial or financial relationships that could be construed as a potential conflict of interest.
