# Supplementary material for: Entrainment and Synchronization to Auditory Stimuli During Walking in Healthy and Neurological Populations: A Methodological Systematic Review
Source: Front Hum Neurosci. 2018 Jun 26;12:263. doi: 10.3389/fnhum.2018.00263 (PMC6028729; doi:10.3389/fnhum.2018.00263)
Supplement: Supplementary file 1 [file Data_Sheet_1.docx]

The supplementary table: STROBE quality checklist

Appendix 1. Search strategy

Supplementary table. STROBE quality checklist

| STROBE statement | | 1 | 2 | 3 | 4 | 5 | 6 | 7 | 8 | 9 | 10 | 11 | 12 | 13 | 14 | 15 | 16 | 17 | 18 | 19 | 20 | 21 | 22 |
| --- | --- | --- | --- | --- | --- | --- | --- | --- | --- | --- | --- | --- | --- | --- | --- | --- | --- | --- | --- | --- | --- | --- | --- |
| Buhmann et al., 2016 | N Y | | Y | Y | Y | Y | N | Y | Y | N | N | Y | Y Y Y Y | N N N | N Y | Y | Y Y N | Y | Y | Y | Y | Y | Y |
| Cha et al., 2014 | N N | | Y | Y | Y | Y | Y | Y | Y | N | N | Y | Y N N Y | N N N | Y Y | Y | Y N N | N | N | N | Y | Y | N |
| Dickstein, Plax, 2012 | N Y | | Y | Y | Y | N | N | N | N | N | N | N | Y Y N N | N N N | Y N | Y | Y N N | N | Y | N | Y | Y | N |
| Dotov et al., 2016 | N Y | | Y | Y | Y | Y | Y | Y | Y | N | N | Y | Y Y N Y | N N N | Y N | Y | Y Y N | Y | Y | Y | Y | N | Y |
| Hove et al., 2012 | N Y | | Y | N | Y | Y | Y | Y | Y | N | N | Y | Y Y N N | N N N | N N | Y | Y N N | Y | Y | Y | Y | Y | Y |
| Leow et al., 2015 | N N | | Y | Y | Y | N | N | Y | Y | N | N | Y | Y Y N N | Y Y N | N N | Y | Y N N | Y | Y | N | Y | Y | N |
| Leow et al., 2014 | N Y | | Y | Y | Y | Y | Y | Y | Y | Y | N | Y | Y Y Y Y | Y Y N | N Y | Y | Y Y N | Y | Y | Y | Y | Y | Y |
| Marmelat et al., 2014 | N Y | | Y | Y | Y | N | N | Y | Y | N | N | Y | Y Y N Y | N N N | N N | Y | Y N N | Y | Y | Y | Y | Y | Y |
| McIntosh et al., 1996 | N Y | | Y | N | Y | N | Y | N | Y | N | N | N | N N N N | N N N | N N | Y | Y N N | Y | Y | N | Y | Y | N |
| Mendonça et al., 2013 | N Y | | Y | Y | Y | N | Y | Y | Y | N | N | Y | Y Y N Y | N N N | N N | Y | Y N N | Y | Y | N | Y | Y | Y |
| Nomura et al., 2012 | N Y | | Y | Y | Y | N | Y | Y | Y | N | N | Y | Y Y N N | N N N | N N | Y | Y N N | N | Y | N | Y | N | N |
| Pelton et al., 2010 | N Y | | Y | Y | Y | Y | Y | Y | Y | N | N | Y | Y Y Y Y | Y Y N | Y N | Y | Y N N | N | Y | N | Y | N | Y |
| Roerdink et al., 2009 | N Y | | Y | Y | Y | Y | Y | Y | Y | N | N | Y | Y Y Y Y | Y Y N | Y Y | Y | Y N N | Y | Y | Y | Y | Y | Y |
| Roerdink et al., 2011 | N Y | | Y | Y | Y | Y | N | N | Y | N | N | Y | Y Y Y Y | Y N N | Y N | Y | Y N N | N | Y | N | Y | Y | Y |
| Terrier, Dériaz, 2012 | N Y | | Y | Y | Y | Y | Y | Y | Y | N | N | Y | Y Y N Y | Y Y N | N N | Y | Y N N | Y | Y | Y | Y | Y | Y |
| Thaut et al., 1999 | N Y | | Y | Y | Y | Y | Y | Y | Y | N | N | Y | Y Y N N | Y N N | Y N | Y | N Y N | N | N | N | Y | Y | Y |

Legend. 1-title and abstract; 2- background/rationale; 3- objectives; 4- study design; 5- setting; 6- participants; 7-variables; 8- data sources/measurement; 9- bias; 10- study size; 11- quantitative variables; 12- statistical methods; 13- participants; 14- descriptive data; 15- outcome data; 16- main results; 17- other analysis; 18- key results; 19- limitations; 20- interpretation; 21- generalisability; 22- funding

Appendix 1. Search strategy

1. Synchronization
2. Rhythm OR Pulse OR Music OR Metronome OR Melody OR Beat OR auditory stimuli
3. 1 AND 2
4. Gait OR Walking OR Treadmill Walking OR Indoor Walking OR Outdoor Walking
5. 3 AND 5
